# Pregnancy-associated Spontaneous Pneumomediastinum: A Contemporary Review

**DOI:** 10.7759/cureus.3452

**Published:** 2018-10-15

**Authors:** Narcisse O Amine, Christine M Lomiguen, Asma Iftikhar, Sonu Sahni

**Affiliations:** 1 Department of Primary Care, Touro College of Osteopathic Medicine, New York, USA; 2 Department of Anatomy, Touro College of Osteopathic Medicine, New York, USA; 3 Department of Pulmonology, New York Presbyterian Hospital Queens, New York, USA; 4 Department of Internal Medicine, Brookdale University Hospital Medical Center, New York, USA

**Keywords:** spontaneous pneumomediastinum, pneumomediastinum, pregnancy, labor, subcutaneous emphysema, dyspnea, chest pain

## Abstract

Spontaneous pneumomediastinum (SPM), also referred to as mediastinal emphysema, is defined as the presence of free air in the mediastinal cavity without a clear and identifiable cause. Spontaneous pneumomediastinum, in general, is a relatively rare condition, more so in the setting of pregnancy or labor. Clinically, SPM may present as dyspnea, chest pain, and subcutaneous swelling, which may be of serious concern in the setting of pregnancy. A comprehensive literature review revealed that the majority of patients are primiparas, of a younger age, and have term or longer durations of pregnancy. The second stage of labor was found to be most commonly associated with the development of SPM. The pathomechanism suggests that performing the Valsalva maneuver during the active stages of labor may play a role in the development of SPM. Once diagnosed, patients with SPM in pregnancy are admitted to the hospital, treated conservatively, and followed until resolution. SPM must be diagnosed and managed promptly due to rare but serious complications. In addition, dyspnea or chest pain with an unknown etiology should include SPM in the differential diagnosis, especially in the setting of pregnancy and labor.

## Introduction and background

Introduction

Pneumomediastinum (PM) is defined as the presence of free air in the mediastinal cavity, which was originally described by Laënnec in 1819 [1]. Pneumomediastinum is most commonly seen secondary to a known cause, such as blunt force trauma or iatrogenic in nature (i.e. endoscopic procedures or central line placement). However, PM without a clearly defined etiology is termed spontaneous pneumomediastinum (SPM). SPM, though with the moniker “spontaneous,” may occur due to various physiologic or pathologic processes. It is most often seen in the setting of underlying pulmonary conditions, such as asthma, interstitial lung disease, or chronic tobacco use [2]. A physiologic process in which SPM may occur is in the setting of pregnancy, labor, and delivery. Though it is a rare occurrence, it may indicate other, possible, serious underlying pathologies. Its clinical presentation is also often confused with that of other chronic pulmonary conditions and may remain undiagnosed. Herein, the authors attempt to summarize the entity of SPM in the setting of pregnancy through a case and literature review. The goal is to form an awareness of the clinical presentation and course of SPM in pregnancy. The findings of our comprehensive review are presented along with a review of the literature outlining the clinical presentation, diagnostic algorithm, as well as the therapeutic process in the setting of SPM.

Methods

A search was conducted of the National Library of Medicine’s MEDLINE/PubMed databases, with the objective of identifying all articles published in the English language between January 1980 and May 2018 with “spontaneous pneumomediastinum” or “mediastinal emphysema” in conjunction with “pregnancy” or “labor.” Combinations of medical subject heading terms associated with spontaneous pneumomediastinum in pregnancy or labor were also searched, including “postpartum pneumomediastinum,” “peripartum pneumomediastinum,” and “obstetric mediastinal emphysema.” We mainly selected publications that were recently published but did not exclude any relevant older manuscripts. We also searched the reference lists of all articles identified by this search strategy and selected those judged to be relevant. All pertinent literature was retrieved, analyzed, and thoroughly searched in order to identify any potential additional manuscripts that could be referenced. All data were accessed between January and June 2017. Our comprehensive PubMed/MEDLINE search revealed a total of 184 manuscripts, of which duplicates, articles not of the English language, or not related to our focus were excluded. This yielded a total of 44 manuscripts that were completely assessed and incorporated into this review.

## Review

Pregnancy and pneumomediastinum

**Figure 1:**
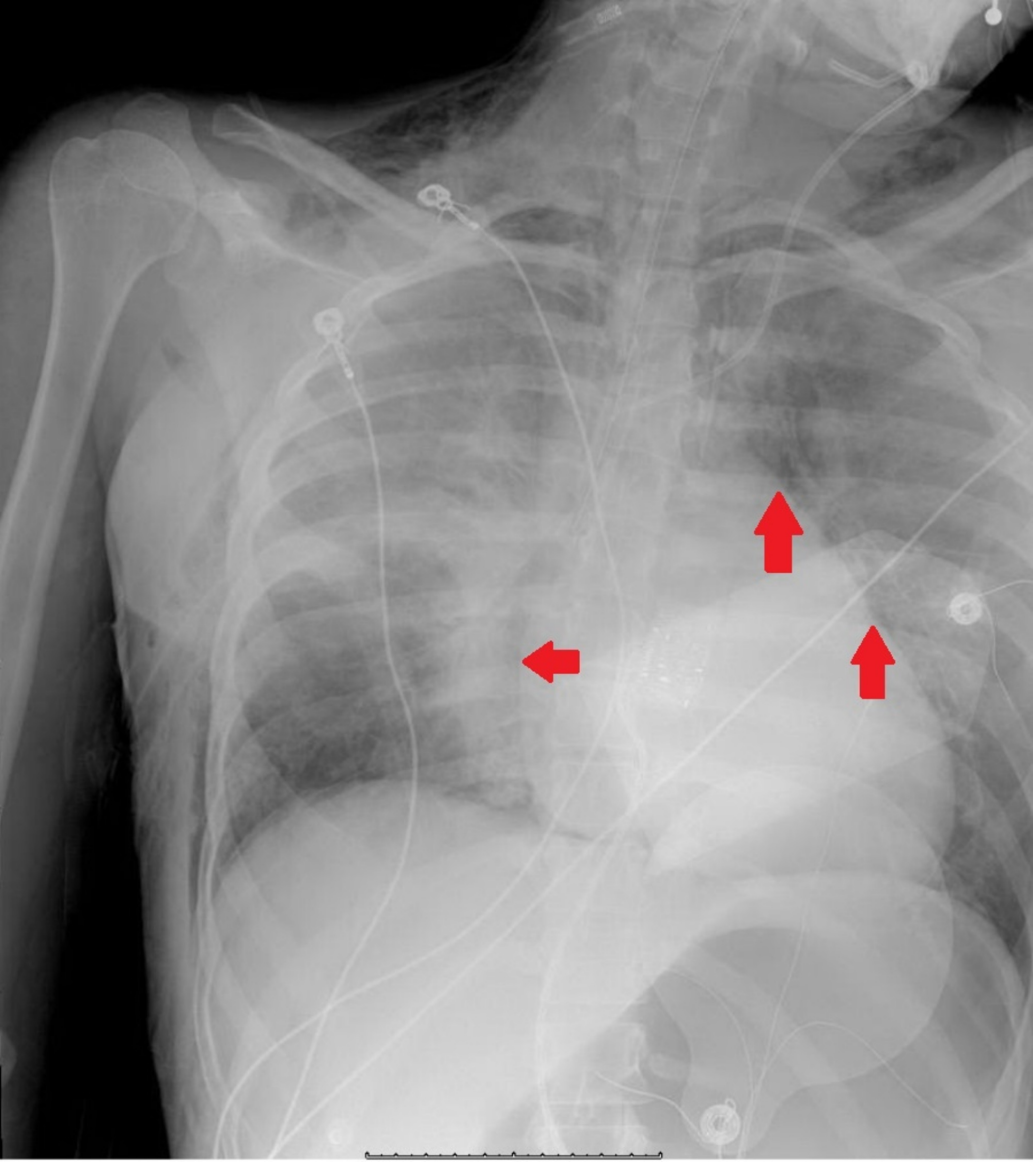
Chest X-ray showing the presence of pneumomediastinum Red arrows point to free air around the heart silhouette.

**Figure 2:**
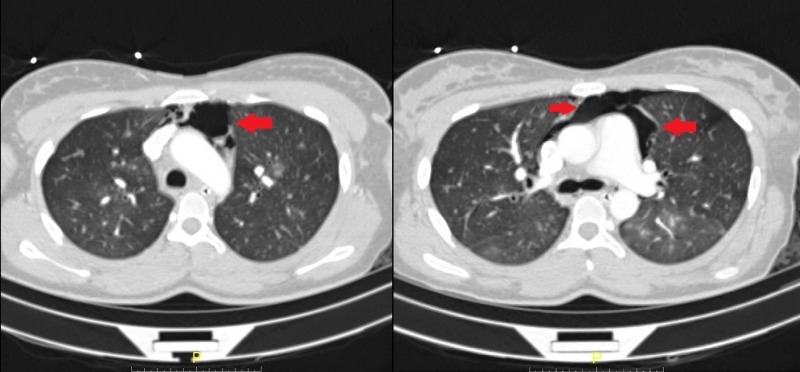
Computer tomography showing the presence of free air in the mediastinum anterior to the heart and great vessels Red arrows point to the pockets of free air in the mediastinal cavity.

The mediastinal cavity is delineated laterally by the pleural cavities, inferiorly by the diaphragm, superiorly by the thoracic inlet, anteriorly by the sternum, and posteriorly by the thoracic vertebrae. The structures encompassed in the mediastinum are primarily cardiac in nature, including the heart, pericardium, as well as the great vessels. Pneumomediastinum refers to the presence of free air within the mediastinal cavity. SPM, initially described by Laennec in 1819, was further characterized in a case series by Hamman in 1939 [3]. Radiologic findings have been shown in Figures 1-2. Spontaneous pneumomediastinum, in general, is a relatively rare condition with a reported incidence of less than 1:44,000 and in the setting of pregnancy or labor, approximately 1:100,000 [4-5]. Though many pre-existing medical conditions have been thought to predispose to the development of SPM, the pathophysiologic process is the same [2].

Theory has it that sudden changes in intrathoracic pressure may precede the development of SPM. The pathogenesis of SPM was first proposed by Macklin in 1939. His proposed mechanism was that alveolar ruptures lead to air dissection along bronchovascular sheaths with the eventual spreading of this inspired air into the mediastinum [6]. This was later coined the "Macklin Effect" in 1944, which broke down the formation of PM into a linear process beginning with alveolar ruptures, leading to air dissecting along bronchovascular sheaths and culminating with the spread of interstitial emphysema into the mediastinum [7]. In the setting of pregnancy, it has been theorized that the Valsalva maneuver produced associated with the second stage of labor may cause a rupture of the alveoli leading to this condition and the propagation of the Macklin effect.

There are four stages of labor. The first stage lasts from the onset of true labor to complete dilation of the cervix. The second stage spans from a complete dilation of the cervix to the birth of the baby. The third stage lasts from the birth of the baby to the delivery of the placenta. The fourth stage spans from the delivery of the placenta to the stabilization of the patient's condition, usually at about six hours postpartum [8]. SPM is most often seen in the second stage of labor and does not negatively affect the following pregnancies. This may due to the “pushing” and strain faced in this stage of labor.

Pathophysiology

**Figure 3:**
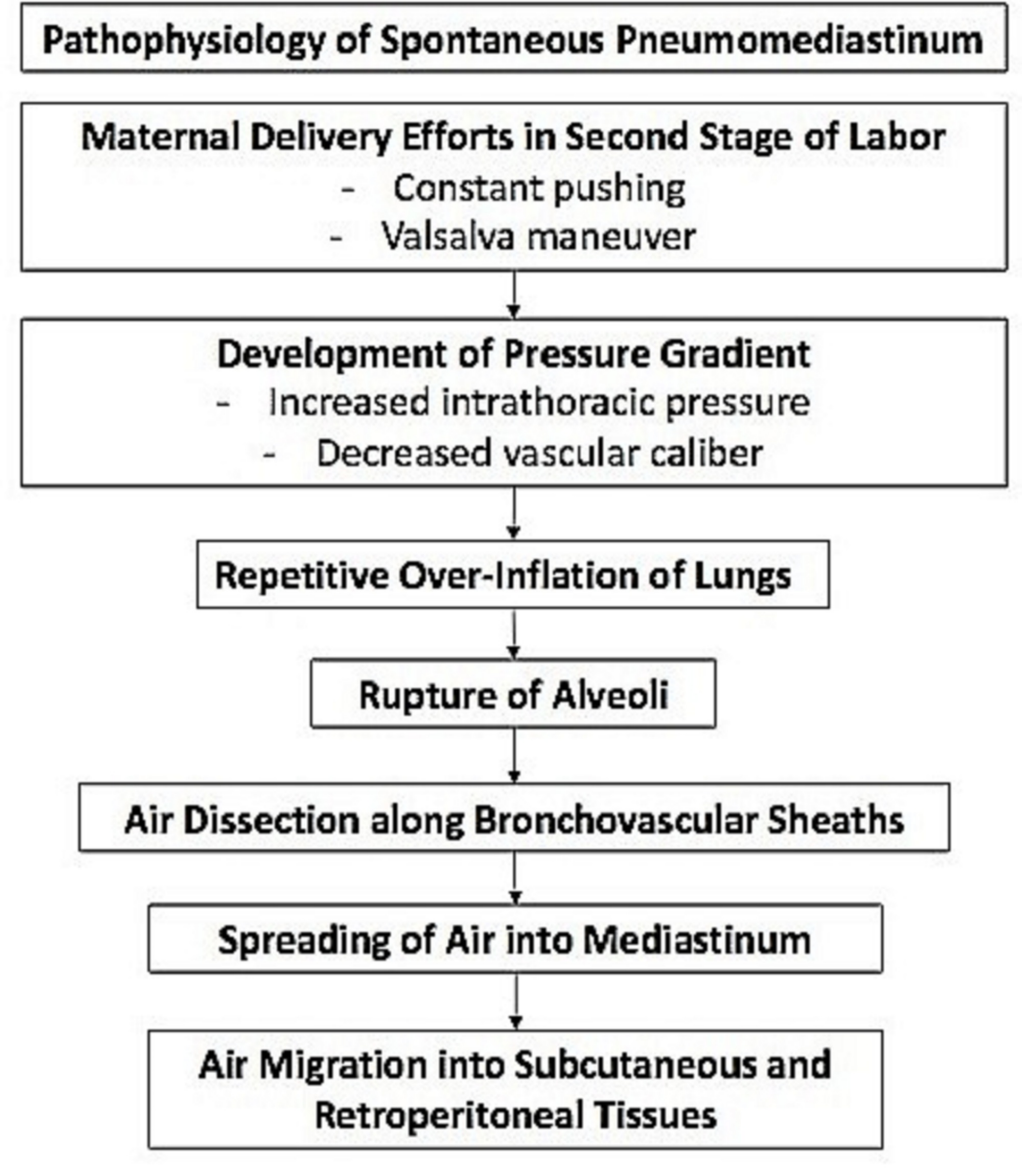
The proposed pathophysiologic process of spontaneous pneumomediastinum in pregnancy

As mentioned, the Valsalva maneuver has been implicated in the development of SPM. The Valsalva maneuver is a forced expiratory effort against a closed glottis, which results in a change in intrathoracic pressure, affecting venous return, cardiac output, arterial pressure, and heart rate. During Valsalva, the intrathoracic pressure becomes positive due to the compression of the thoracic organs. Of the various explanations for pneumomediastinum in pregnancy, the most widely accepted theory implicates the rupture of marginal pulmonary alveoli as a result of repetitive overinflation of the lungs and of high intra-alveolar pressures during the second stage of labor [9]. In maternal delivery efforts, constant pushing during labor consequently results in increased intrathoracic pressure in the presence of decreased vascular caliber; this establishes a pressure gradient in the vascular sheath, which allows air to dissect into the mediastinum. From the mediastinum, air migrates along the fascial planes into the subcutaneous and retroperitoneal tissues [10-11]. An outline of this process has been shown in Figure 3 [12].

In addition to the “pushing” associated with the delivery of the fetus, other physiological alterations of the respiratory system occur during the latter parts of pregnancy. These changes are mainly the consequence of the progestin stimulation of the respiratory drive and consist of a reduction in the functional residual capacity and an increase of about 70% in alveolar ventilation due to a breathing pattern with augmented respiratory rate and tidal volume. This may also contribute to the development of SPM [12]

Clinical characteristics

Our literary search methodology resulted in a total of 34 cases of pregnancy or labor-related spontaneous pneumomediastinum, which have been presented in Table 1 [5,13-41]. From our results, we have focused on certain patient characteristics, such as the duration of the pregnancy, parity, smoking history, as well as presenting signs and symptoms. SPM in pregnancy is typically seen in young patients; of the cases we have examined, the average age was 23.4 +/- 5.7 years of age. The majority of patients were nonsmokers without a previous history of asthma, showing that the diagnosis can be designated as “spontaneous” due to the unclear etiology.

**Table 1:** Summary of spontaneous pneumomediastinum cases in pregnancy

Author	Age	Parity	Stage of Labor	Duration of Labor (hours)	Duration of Pregnancy (weeks)	Treatment
Crean et al. 1981 [5]	22 y/o	1^st^	2^nd^ stage	12	N/A	Oxygen
Hubbert et al. 1981 [13]	17 y/o	N/A	2^nd^ stage	N/A	N/A	Oxygen, fentanyl, diazepam, & succinycholine
	18 y/o	N/A	2^nd^ stage	N/A	N/A	Oxygen
Karson et al. 1984 [14]	20 y/o	1^st^	1^st^ stage	N/A	9	Pyridoxine 2x daily
	20 y/o	1^st^	1^st^ stage	N/A	42	None
Jensen et al. 1987 [15]	24 y/o	1^st^	2^nd^ stage	10.5	41	Oxygen
Ramirez-Rivera et al. 1990 [16]	15 y/o	1^ST^	2^nd^ stage	14	N/A	Oxygen
Jayran-Nejad et al. 1993 [17]	18 y/o	1^st^	2^nd^ stage	15.6	42	Analgesia
Seidl et al. 1994 [18]	23 y/o	2^nd^	N/A	N/A	42	None
Gocmen et al. 1997 [19]	17 y/o	1^st^	N/A	11	39	Symptomatic management & monitoring
Shyamsunder et al. 1999 [20]	18 y/o	2^nd^	2^nd^ stage	N/A	6	None
Gorbach et al. 1997 [21]	21 y/o	N/A	2^nd^ stage	4	9	IV Fluids, Promethazine
Raley et al. 1997 [22]	N/A	N/A	N/A	N/A	41	Oxygen
Dhrampal et al. 2001 [23]	36 y/o	N/A	2^nd^ stage	N/A	37	Oxygen & analgesia
Sutherland et al. 2002 [24]	32 y/o	1^st^	2^nd^ stage	8	N/A	None
	22 y/o	1^st^	2^nd^ stage	13	N/A	None
Miguil et al. 2004 [25]	19 y/o	N/A	2^nd^ stage	N/A	40	Oxygen & analgesia
Balkan et al. 2006 [26]	25 y/o	1^st^	2^nd^ stage	N/A	36	Oxygen
Bonin et al. 2006 [10]	27 y/o	1^st^	2^nd^ stage	6	38	Lorazepam for anxiety; anxiolytics for dyspnea
North et al. 2006 [27]	32 y/o	N/A	2^nd^	N/A	N/A	Laxatives
Yadav et al. 2008 [28]	21 y/o	1^st^	2^nd^ stage	1.3	N/A	Oxygen & analgesics
Mahboob et al. 2008 [9]	24 y/o	N/A	2^nd^ stage	N/A	39	Oral antibiotics
Zapardiel et al. 2009 [29]	29 y/o	1^st^	4^th^ stage – only time	7	39	Oxygen
Speksnijder et al. 2010 [30]	15 y/o	N/A	2^nd^ stage	N/A	28	Insulin, fluid, & potassium supplementation
Beynon et al. 2011 [31]	18 y/o	1^st^	2^nd^ stage	4.3	39 + 2	Antibiotics & analgesia
Wozniak et al. 2011 [32]	20 y/o	N/A	2^nd^ stage	N/A	41	Observation
Shrestha et al. 2011 [33]	19 y/o	1^st^	2^nd^ stage	N/A	36	None
Kuruba et al. 2011 [34]	32 y/o	2^nd^	2^nd^ stage	1.5	40	None
McGregor et al. 2011 [35]	27 y/o	1^st^	2^nd^ stage	1.5	40	Oxygen & analgesia
Khoo et al. 2012 [36]	33 y/o	1^st^	2^nd^ stage	12	40	Analgesia & best rest
Kouki et al. 2013 [37]	23 y/o	1^st^	2^nd^ stage	9	40	Oxygen & analgesics and sedatives
Cho et al. 2015 [38]	28 y/o	1^ST^	2^nd^ stage	5	36	Oxygen & analgesics
Scala et al. 2016 [39]	30 y/o	N/A	N/A	N/A	40	None
Nagarajan et al. 2017 [40]	30 y/o	N/A	2^nd^ stage	N/A	41	Observation
Berdai et al. 2017 [41]	22 y/o	1^ST^	2^nd^ stage	2H	40	Oxygen

Parity

Parity was of interest in this patient population, as it has been reported that the second stage of labor is prolonged in a nulliparous woman as compared to a multiparous woman. In a study by Albers et al., it was found that the mean length of the second stage of labor in nulliparas was 54 minutes versus 18 minutes for multiparas [42]. Of the cases we have reviewed and the ones that have provided a parity status, it was found that 20/34 (58.8%) of cases were of women who were primiparas. It appears as if subsequent pregnancies are not as affected, as only 3/34 (8.8%) were observed to be in second pregnancies and beyond.

Pregnancy Duration and Delivery

As the majority of cases described are associated with pregnancy, the authors felt it necessary to analyze the duration of pregnancy among all cases. Cases that did not provide the duration of pregnancy or in the cases of early termination or fetal demise, duration of pregnancy was not considered. Of the cases that were reviewed, it was determined that the average length of pregnancy was 39.04 +/- 2.94 weeks. This is in line with previously reported works that reported that pregnancy-associated SPM most commonly occurs in the setting of full-term vaginal births [5]. Pregnancy-associated SPM is almost always seen in the setting of natural vaginal delivery, the pathophysiology of which has been discussed above. In very few cases, SPM in pregnancy has been seen in the first and fourth stages of labor, with 28 (80%) of cases occurring in the second stage of labor.

Signs and Symptoms

Spontaneous pneumomediastinum, in general, presents with similar signs and symptoms among patients. The most commonly reported symptom is chest pain, followed by dyspnea [2]. Here, we focused on spontaneous pneumomediastinum presented during pregnancy. Of the cases that were reported, a summary of presenting clinical signs and symptoms has been shown in Table 2.

**Table 2:** Clinical signs and symptoms of pregnancy-associated SPM SPM: spontaneous pneumomediastinum

Signs & Symptoms	Number of Cases (%)
Swelling & Subcutaneous Emphysema (face, neck, etc.)	21 (60.0)
Dyspnea	16 (45.7)
Chest Pain	13 (37.1)
Crepitus	10 (28.6)
Tachycardia	7 (20.0)
Vomiting	5 (14.3)
Cough	3 (8.6)

Although not in the situation of pregnant patients, SPM has been reported due to straining exercises, which also involve the Valsalva maneuver, and forceful coughing. It additionally has been reported in patients with a history of asthma, chronic obstructive pulmonary disease (COPD), and upper respiratory infection [2].

Management and complications

**Figure 4:**
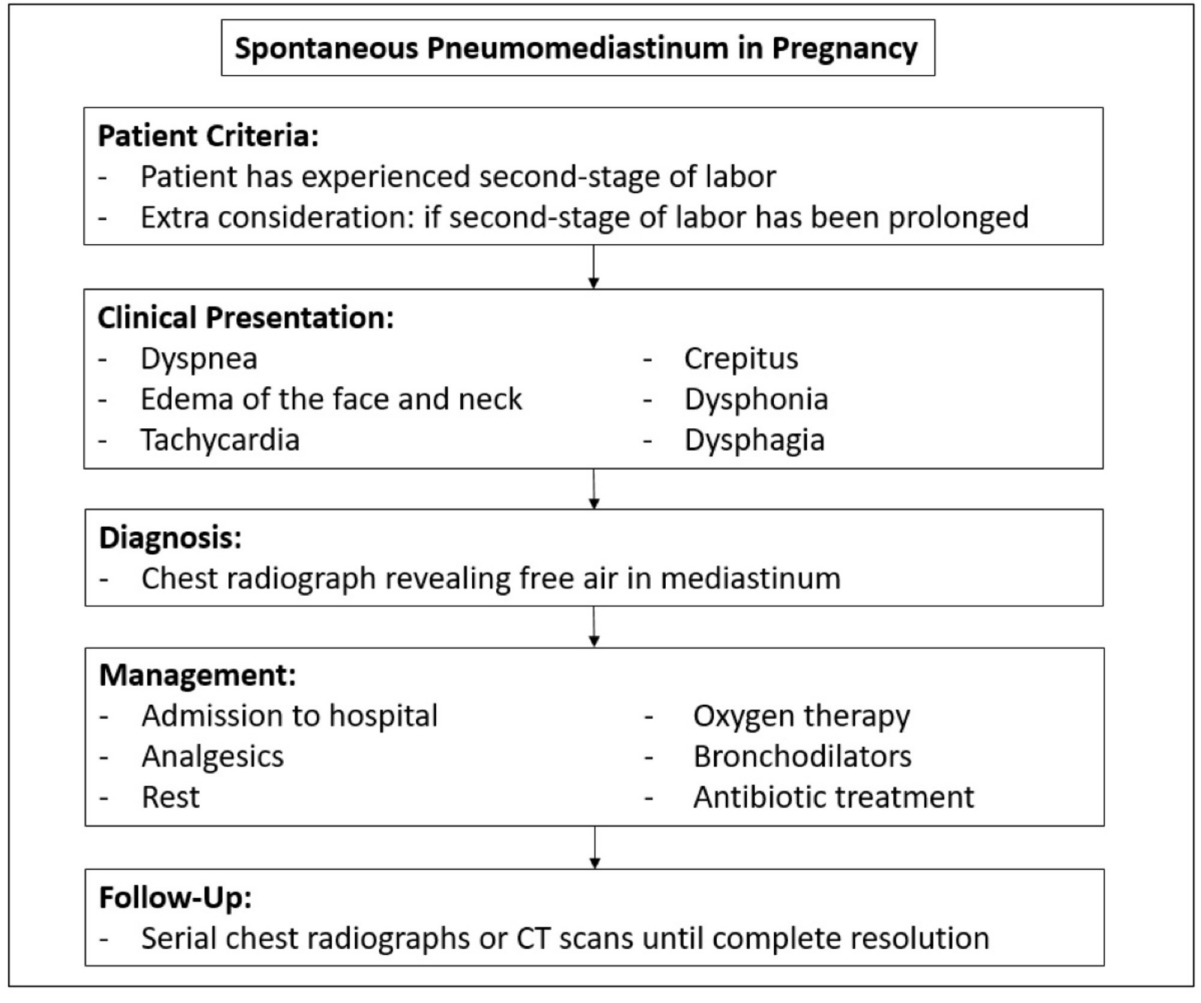
Diagnostic and management algorithm of SPM in pregnancy SPM: spontaneous pneumomediastinum

Spontaneous pneumomediastinum, in general, is not often included in the differential diagnosis and even more so in the setting of pregnancy due to its vague presentation. It is often treated as another causative factor of chest pain, dyspnea, and wheezing, such as asthma exacerbation. SPM usually follows a benign course and management is often conservative. The majority of patients admitted to the hospital with a diagnosis of SPM are treated with analgesics, rest, oxygen therapy, bronchodilators, and occasionally antibiotic treatment [2,43]. If any pre-disposing condition (asthma, infection, airway obstruction, etc.) is responsive to pharmacological management, then SPM is self-limiting [2,44]. A diagnostic algorithm has been provided in Figure 4.

Complications of SPM are rare as the diagnosis is often expeditious due to an often overly ominous presentation. Timely management is important to avert any serious complications. If untreated or not monitored, SPM has shown a possibility to convert to tension or malignant PM, which may lead to a compression of the great vessels, ultimately having a deleterious effect on the fetus in the setting of pregnancy [7]. Mortality is extremely rare, as it is known that spontaneous pneumomediastinum typically resolves quickly by the free air being absorbed into the surrounding tissue. In the 34 cases we observed, there were no reports of complications and all cases were managed conservatively.

## Conclusions

Spontaneous pneumomediastinum is a rare occurrence in the physiologic setting of pregnancy, labor, and delivery. It is thought the Valsalva maneuver produced in natural vaginal delivery and its physiologic consequences are the impetus for the development of SPM. A review of case reports in the literature revealed that a majority of patients are primiparas, of a younger age, and have term or longer durations of pregnancy. As the literature suggests, the second stage of labor is most commonly associated with the development of SPM. The cases reviewed are associated with the most common clinical signs and symptoms of SPM such as chest pain and dyspnea. Once diagnosed, patients should be admitted to the hospital, monitored, treated with analgesics, rest, and oxygen therapy until complete or near resolution. Once released from the hospital, patients may be followed with serial chest radiographs or CT scans until complete resolution. Physicians need to be aware of the possibility of SPM in parturient patients and include SPM in the differential, especially in the setting of dyspnea, chest pain, and relevant physical findings.
